# Quit4baby: Results From a Pilot Test of a Mobile Smoking Cessation Program for Pregnant Women

**DOI:** 10.2196/mhealth.3846

**Published:** 2015-01-23

**Authors:** Lorien C Abroms, Pamela R Johnson, Christina L Heminger, Judith M Van Alstyne, Leah E Leavitt, Jennifer M Schindler-Ruwisch, Jessica A Bushar

**Affiliations:** ^1^Milken Institute School of Public HealthPrevention & Community HealthThe George Washington UniversityWashington, DCUnited States; ^2^Voxiva, Inc.Washington, DCUnited States; ^3^National Healthy Mothers, Healthy Babies CoalitionAlexandria, VAUnited States

**Keywords:** mobile health, tobacco cessation, pregnancy, text messaging

## Abstract

**Background:**

Text messaging (short message service, SMS) programs have been shown to be effective in helping adult smokers quit smoking. This study describes the results of a pilot test of Quit4baby, a smoking cessation text messaging program for pregnant smokers that was adapted from Text2quit.

**Objective:**

The study aimed to demonstrate the feasibility and acceptability of Quit4baby for women currently enrolled in Text4baby, a perinatal health text messaging program.

**Methods:**

Pregnant women enrolled in Text4baby and who were current smokers or had quit within the last 4 weeks (n=20) were enrolled in Quit4baby. Those under the age of 18, not pregnant, not current smokers, those using nicotine replacement therapy, and those not interested in participating were ineligible. Participants were surveyed at baseline and at 2 and 4 weeks postenrollment.

**Results:**

Most participants responded to the program favorably. Highly rated aspects included the content of the program, skills taught within the program, and encouragement and social support provided by the program. Participants reported that the program was helpful in quitting, that the program gave good ideas on quitting, and that they would recommend the program to a friend. Suggestions for improvement included increasing the message dose and making the quitpal more interactive.

**Conclusions:**

This pilot test provides support for the feasibility and acceptability of Quit4baby. Future studies are needed to assess whether Quit4baby is effective for smoking cessation during pregnancy.

## Introduction

Cigarette smoking in pregnancy poses serious health risks to both the woman and the fetus. It has been shown to cause adverse fetal outcomes, including stillbirths, spontaneous abortions, premature births, low birth weight, and sudden infant death syndrome (SIDS), and has been linked to cognitive and behavioral problems in children [1]. It is estimated that greater than 20% of low-birth-weight births could be prevented by eliminating smoking during pregnancy [2].

Among the general population of adult smokers, pregnant smokers in the United States are typically younger, less educated, and more likely to be white or of Native American ancestry [3]. They are twice as likely to be on Medicaid, the government-sponsored insurance program for low-income Americans [4,5]. About 45% of pregnant smokers are able to quit during their pregnancy [6]. Barriers to quitting include a lack of willpower, limited access to cessation services, stigma, stressful life events and relationships, and smoking among family and friends [7]. Currently, many pregnant smokers do not receive recommended smoking cessation counseling [8]. Pregnant smokers are less likely than non-pregnant smokers to call quitlines, and uptake of programmatic offerings aimed at pregnant smokers is low [8]. In addition, medications that are effective for smoking cessation and a staple in treatment programs are not recommended in the US for use among pregnant women [2].

In the US, 85% of all adults have mobile phones [9] and 72% of mobile phone owners send and receive text messages (short message service, SMS) [10]. Among 18- to 29-year-old women (the group most likely to be childbearing), 96% own a mobile phone and 95% send and receive text messages [9]. Texting is also more common in people with Medicaid health insurance compared with other forms of private insurance [11]. Text message-based smoking cessation programs have been found to increase abstinence among adult smokers [12-14].

Mobile phone smoking cessation programs may be especially promising with pregnant women because of the almost universal penetration of mobile phone messaging in this population [9], and because services can be received anonymously, reducing the effect of stigma as a barrier to help seeking [15]. To date, few text messaging studies have been conducted on pregnant smokers [16,17], and existing studies consist of pilots with mixed results. One pilot study of an SMS text-based trial with pregnant smokers found that most women read most texts received. The study also found that women receiving scheduled, gradual reduction texts had a higher rate of cotinine-confirmed, 7-day point prevalence at the end of pregnancy and greater reductions in smoking compared with those who received supportive texts [16]. Another study of pregnant smokers in the United Kingdom who were offered a text messaging program in conjunction with a tailored brochure found that the program modified intentions to quit and resulted in higher levels of self-efficacy for quitting and beliefs about the harms of smoking. However, at a 3-month follow-up, the intervention group showed no difference in 7-day point prevalence or cotinine-confirmed abstinence, regardless of baseline differences in prenatal smoking history [17].

The current study was aimed at demonstrating the feasibility and acceptability of Quit4baby, a smoking cessation text messaging program for pregnant smokers in the US. This program was novel for two reasons. First, unlike previous programs, it was developed from an existing program that has been previously studied in adult smokers and proven to work [13]. Text2quit, a smoking cessation text messaging program for adults, has been shown to increase biochemically confirmed quit rates in adult smokers [13,18]. Second, this program was designed around a large, existing service for pregnant women in order to maximize its potential to reach large numbers of pregnant smokers. Quit4baby was designed to serve as a potential add-on service for Text4baby, an existing national texting program for pregnant women that provides perinatal health information, and has enrolled over 800,000 users since its launch in 2010 [19]. Text4baby has been shown to increase healthy beliefs and attitudes related to taking prenatal vitamins, visiting a health care provider, and avoiding alcohol during pregnancy [20-21]. Given the large subscriber base of Text4baby, Quit4baby may have the potential to increase the reach of smoking-cessation programs that are specifically targeted to pregnant smokers.

Specifically, this study reports on participants’ experiences with the program after a 4-week trial. Areas of interest include their overall rating of the program, their level of engagement and use of the interactive features, and the types of interactions that were favored.

## Methods

### Procedures and Sample

Recruitment was conducted between December 17, 2013 and January 31, 2014, after the study received Institute Review Board (IRB) approval from the George Washington University (GW). Recruiting took place through a broadcast text message sent to all Text4baby subscribers less than 30 gestational weeks pregnant and living in the states of Pennsylvania, Maryland, West Virginia, North Carolina, Kentucky, and Tennessee (n=4450). These states were selected because of their moderate to high prevalence of smoking during pregnancy [22]. The text recruitment message was as follows: “Hi Mom! We are working to make Text4baby better. May we call and talk with you at this number about being part of a study? Reply 1 for Yes, 2 for No.” Subscribers who replied 1 (Yes) were notified via SMS text message that they would be called.

Of the 4450 women contacted by text message, 409 (9.19%) responded that they were interested in being part of a study. GW research staff called all interested subscribers, reached 120 women and, of those, 20 (16.7%) were found to be eligible. Text4baby subscribers were eligible if they were aged 18 years or older, a current smoker or had quit within the last 4 weeks, and currently pregnant. Reasons for not being eligible for the study included age (less than 18) (4/100, 4.0%) and smoking status (96/100, 96.0%).

All eligible participants agreed to enroll in the study. GW research staff administered the baseline survey over the phone and enrolled participants in Quit4baby. All participants were asked by GW research staff to set a quit date within the next 2 weeks. Once enrolled, participants activated the program via SMS text messaging and began receiving Quit4baby program messages, while continuing to receive Text4baby messages at the usual frequency (3 messages per week). Follow-up phone surveys were conducted at 2 and 4 weeks postenrollment. Participants were sent a $25 gift card for each survey completed.

### Intervention

**Figure 1:**
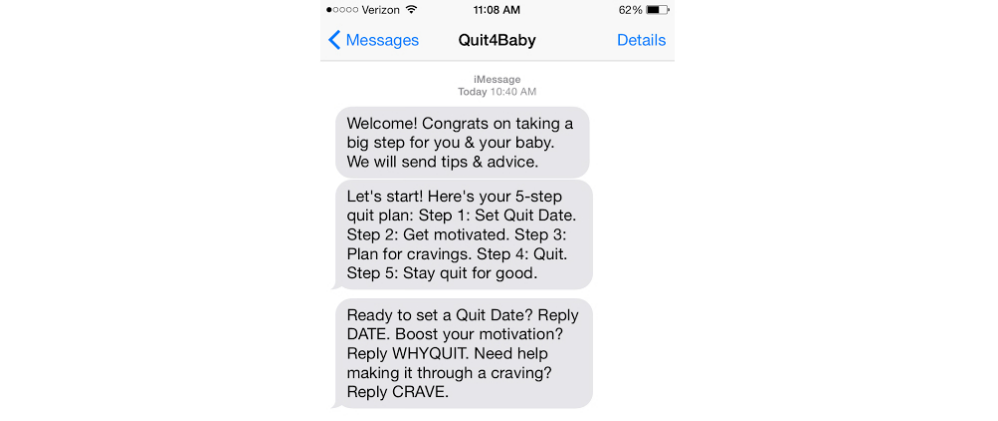
Quit4Baby screenshot.

Quit4baby 1.0 was developed by modifying Text2quit, a proven smoking-cessation text messaging program [13,18], in order to adapt content and tailor the program to the context of pregnancy and to be consistent with the US Public Health Service Clinical Practice Guideline [2] (Figure 1). Like Text2quit, Quit4baby was based on the Social Cognitive Theory [23] with messages aimed at improving self-efficacy for quitting (with encouragement and motivational messages), describing outcome expectations from quitting, increasing social support for quitting (via the quitpal), enabling vicarious learning through the modeling of effective quitting strategies and coping skills (with keyword TIP and the quitpal), increasing behavioral capability for quitting (with keyword CRAVE, a quit plan, quit date, and interactive support), and regularly recommending calling a quitline.

Content for Quit4baby 1.0 was developed by GW with input from Voxiva and the National Healthy Mothers, Healthy Babies Coalition, and was reviewed by representatives from the Text4baby Content Council and an expert pregnancy smoking cessation consultant. Messages were developed to be consistent with the US Public Health Service Clinical Practice Guideline for smoking cessation during pregnancy [2]. There were six changes made to Text2quit to develop Quit4baby 1.0.

1. The medication protocols from Text2quit were suspended—all mentions of medications, including nicotine replacement therapy (NRT), were removed from the messages, consistent with clinical guidelines [2].

2. The language in the messages was revised to recognize that all enrollees were pregnant and to include pregnancy-specific information, such as the harms of smoking on mother and baby, as this type of educational messaging is clinically recommended to encourage cessation among pregnant women [2]. Messages were revised to include reminders about quitting for the baby’s health, as well as the mom’s health, in addition to other information about a healthy pregnancy.

3. The peer ex-smoker, quitpal, was changed to a woman who quit smoking while pregnant.

4. The daily tracker was changed so that participants did not have a specific preset goal for cutting down, as is the case in Text2quit, and participants were asked to only track their smoking in the prequit period as a way of self-monitoring.

5. The program period was shortened to 43 days after enrollment to fit the period of the pilot test.

6. The default date to quit was set to the following Monday, based on research that this may be an appropriate day each week to encourage quitting [24].

Participants received 1 to 5 text messages per day, with the highest dose of text messages sent on their quit date and on the days immediately preceding and following that date. The texts were sent out around three main message protocols: prequit, postquit, and not-quit. Most messages originated from the Quit4baby program, though 12 text messages originated from a fictitious peer female ex-smoker quitpal who had quit during pregnancy and who offered evidence-based advice on quitting. The characteristics of, and messages sent by, the role model were based on real-life experiences of pregnant women. Participants were not told that the quitpal was fictitious, although many assumed she was. Participants were assumed to have quit on their chosen quit date unless they replied to a message to say that they had not quit. In this case, participants were prompted to reset their quit date and if they were not ready to do so, they were routed into the not-quit protocol where they were regularly reminded of the benefits of quitting smoking for mom and baby and urged to set a quit date.

Quit4baby pilot participants had the opportunity to text keywords to the program for additional support. Keywords included WHYQUIT (sends messages about what motivated others to quit), DATE (allows users to reset quit date), CRAVE (provides distractions to get users through craving period), TIP (provides tips on abstaining from smoking), GAME (sends users a trivia game to get through a craving period), REASONS (reminds users of their chosen reasons for quitting), SMOKED (helps users get back on track if they slip or relapse midprogram), and STOP (allows users to end the receipt of the text messaging program). Although these same keywords were used in Text2quit, actual messages were modified to reflect the pregnant status of the participants.

### Measures and Analysis

Measures for this study were collected in the baseline, 2-week and 4-week postenrollment telephone surveys. The baseline survey captured demographic information, text messaging habits, smoking behaviors, beliefs about smoking and quitting, and needs and motivations for quitting while pregnant. Responses to items on the baseline survey, such as selected quit date and reasons for wanting to quit, were used to tailor the content of the text messages within Quit4baby. Nicotine dependence was measured on the baseline survey with the Fagerstrom Test for Nicotine Dependence (FTND) [25].

Participant demographic traits, smoking traits, self-efficacy, and smoking outcomes were also examined in a closed-ended format. A truncated version of the Attitude-Social Influence-Efficacy Model (ASE) [26-28] was used to assess smoking beliefs, smoking knowledge, and self-efficacy at baseline. Participants were asked to rate a variety of smoking belief statement items about the negative effects of smoking on their health and the health of their baby. These statements were rated on a 5-point Likert scale from *completely disagree* (1) to s*trongly agree* (5). Participants were also asked to rate their confidence in their ability to quit smoking while pregnant. These statements were rated on a 5-point Likert scale from *not at all confident* (1) to *completely confident* (5). Participants were asked to clarify why they rated each question that way to elicit further qualitative and open-ended feedback.

Information on current smoking behaviors, smoking beliefs, and perceptions of how the Quit4baby program fit with the Text4baby messages they currently received were obtained from the 2- and 4-week follow-up telephone interviews. Participant satisfaction with the program was measured by a series of questions in which participants were asked to rate their agreement with statements about the texts (eg, “The program was helpful in getting me to try to quit,” “The program gave me good ideas on how to quit,” “I would recommend the program to a friend interested in quitting.”) These statements were rated on a 5-point Likert scale from c*ompletely disagree* (1) to s*trongly agree* (5).

Participants were also asked to make suggestions for improving the program and to note which features they liked and disliked. The majority of questions were closed-ended, but several open-ended questions that elicited qualitative feedback were also elicited. Some open-ended probes were used to learn why a participant responded a certain way to a closed-ended question, for example, “Why did you rank (the messages) that way?” and “How would you improve them?” Others questions were purely qualitative in nature, for example, “How do you feel about your ability to stay quit?” and “Can you tell me if there was anything confusing about the texts?” At 2- and 4-week follow-up, participants were asked if they had smoked at all for the past 7 days as a measure of smoking abstinence.

A retrospective review of the computer records of participants was done to characterize the text message engagement of each participant. Each participant was asked about the number of text messages read (*all, most, some, none*) and the number of text message responses was calculated. Responses included replies to interactive text message surveys, for example, when a participant texts Yes or No after receiving a text which says, “Please be honest, did you quit today?” Responses also included unsolicited requests for help with quitting via keyword, for example, when a participant texts CRAVE for help with a craving. Also of interest was the timing of responses in relation to enrollment and the quit date, for example, “Is there anything you would like to change about the timing of the messages?”, as well as the types of responses that were most used by participants, for example, “Which keywords did you text to the system?” and “Why did or didn’t you initiate conversations with the system?”

Descriptive statistics were used to determine the demographic profile and smoking history of the pilot participants, as well as markers of program engagement, user satisfaction, and recommendations for future improvements. Quantitative analyses were performed using Microsoft Excel 2010 and qualitative analyses were housed in Microsoft Excel 2010 and analyzed by a thematic analysis.

## Results

### Participant Demographics

A total of 20 women enrolled in the Quit4baby pilot program and completed the baseline survey. Of the 20 enrolled, 16 completed the 2-week follow-up survey (80% response rate), and 13 completed the 4-week follow up survey (65% response rate). Basic demographic and smoking characteristics of participants at baseline are indicated in Table 1.

**Table 1:** Participant demographics and smoking characteristics.

Demographic and smoking characteristics of participants^a^ (n=20)		Mean (SD) or n (%)
Age in years, mean (SD)		28.1 (6.1)
**Race, n (%)**		
	White	13 (65)
	Black	5 (25)
	More than one race	2 (10)
**Educational attainment, n (%)**		
	Some high school	3 (15)
	High school degree, technical or trade school	10 (50)
	Some college	4 (20)
	College graduate	3 (15)
**Employment status, n (%)**		
	Employed full time	2 (10)
	Employed part time	4 (20)
	Not employed	14 (70)
**Marital status, n (%)**		
	Single	10 (50)
	Separated	1 (5)
	Partnered	7 (35)
	Married	2 (10)
Children status, n (%)		
	Has other children	14 (70)
**State of residence, n (%)**		
	Pennsylvania	7 (35)
	Tennessee	5 (25)
	North Carolina	4 (20)
	Kentucky	2 (10)
	Maryland	1 (5)
	West Virginia	1 (5)
Smoking status, n (%)		
	Smoked in past 7 days	19 (95)
Average number of cigarettes per day, mean (SD)		7.2 (4.9)
**Time to first cigarette after waking, n (%)**		
	Within 5 minutes	5 (25)
	6-30 minutes	8 (40)
	31-60 minutes	4 (20)
	After 60 minutes	3 (15)
**Believe or strongly believe smoking, n (%)**		
	...is bad for my own health	19 (95)
	...is sociable	18 (90)
	...makes my baby weigh less	18 (90)
	...makes my baby smaller	17 (85)
	...is soothing	8 (40)
	...tastes good	1 (5)

^a^Pregnant women aged 18 and older.

### Rating and Perceptions of the Quit4baby Program

As shown in Table 2, participants gave overall high ratings to the program. Participants agreed and completely agreed that the program was helpful in quitting, that the program gave good ideas on quitting, and that they would recommend the program to a friend. One participant indicated that she found the texts helpful and supportive: “Texts were very helpful...gave (me) extra support.” Another participant reported that the messages contained good ideas on how to quit: “They had ideas I didn’t know about before.”

Participants rated programmatic messages and the following message categories were deemed most helpful: messages that asked them to track their smoking, messages that came from a quitpal, and messages that promoted behavioral substitutions (eg, core messages aimed at providing alternative healthy behaviors to replace smoking, or responses to participant-input TIP keyword). One participant commented on the tracking suggestions in the messages, saying, “(the texts) held me accountable.” Participants called the quitpal, “personal,” “a friend,” and stated that, “It just helps to know someone’s there.” However, one participant felt the messages were a trigger: “Getting this message makes we want to smoke.” Another participant wanted more detail: “(There) was nothing specific in the message.”

Message frequency is an important characteristic of any SMS text message behavior-change program. Out of the 16 pilot participants who responded at the 2-week follow-up about the number of messages received, 14 (88%) felt they received “just the right number,” while 2 (12%) felt they received “too few” messages. No participants reported receiving too many messages. Moreover, pilot participants felt that the Text4baby program messages and the Quit4baby program messages “fit well together” (12/16, 75%), or at a minimum, “fit ok together” (3/16, 19%). Only 1 participant out of 16 (6%) felt the messages from the two programs did not fit well together.

Participants were also asked to share what they liked and did not like about the Quit4baby program. These were open-ended questions that allowed for multiple comments from each individual. Participants most commonly reported liking the content, the skills the program helped them develop, the encouragement the program gave, and the constant reminders the program sent about quitting. Specifically, participants mentioned skills related to cravings (eg, “I didn’t realize that just walking and games made a difference to get through cravings,”) and the social support provided (eg, “Makes you feel like you have more support—someone else is going through the same thing.”)

More than half (10/16, 63%) of all responses to questions regarding program dislikes were “nothing.” The next most commonly reported dislike was participants’ wanting more tips and hints for how to quit smoking. Others mentioned wishing the quitpal “Erika” was a real person, or at a minimum, a programmed mechanism that could respond to their text messages, versus only sending unidirectional information. Table 2 displays participants’ ratings and perceptions of the Quit4baby program elements.

**Table 2:** Participant ratings and perceptions of the Quit4baby program at the 2-week follow-up survey.

Category and item (n=16)		Mean (SD) or n (%)
**Program rating,** ^a^ **mean (SD)**		
	Program was helpful in trying to get me to quit.	4.5 (0.6)
	Program gave me good ideas on how to quit.	4.4 (0.9)
	I would recommend the program to a friend interested in quitting.	4.7 (0.7)
**Message category helpfulness rating,** ^a^ **mean (SD)**		
	Tracking	4.5 (0.8)
	Quitpal	4.3 (0.9)
	Behavioral substitution	4.3 (1.2)
	Social support	4.2 (1.2)
	Stress reduction	3.9 (1.2)
Message timing rating,^a^ mean (SD)		
	Messages came at the right time of day.	4.0 (0.8)
**Message frequency, n (%)**		
	Just right	14 (88)
	Too few	2 (12)
	Too many	0 (0)
**Program fit with Text4baby, n (%)**		
	Fit very well	12 (75)
	Fit ok	3 (19)
	Didn’t fit well	1 (6)
**Programmatic likes, n (%)**		
	Content/skills	6 (30)
	Encouragement	3 (15)
	Reminders	3 (15)
	Message tailoring	2 (10)
	Social support	2 (10)
	On-demand help	1 (5)
**Programmatic dislikes, n (%)**		
	Nothing	10 (63)
	Content/info	3 (19)
	Message frequency	1 (6)
	Personal interaction (lack thereof)	1 (6)
	Message timing	1 (6)

^a^Items were ranked on a scale from 1 (completely disagree) to 5 (strongly agree).

### Program Engagement

Table 3 provides an overview of program engagement. At the 2-week follow-up, all participants reported having read all of the text messages that the program sent. On average, participants sent 5.4 (SD 6.6) text message responses, but there was some variability in responses during the program. Participants were actively engaged in the program for an average of 24.2 (SD 17.0) days from their date of enrollment. This average was calculated by subtracting the participant’s enrollment date from their last recorded date of activity (eg, responding to a survey or texting a keyword). Other enrollees may have still been engaging with the program passively by reading SMS text messages. Lastly, more than half of participants remained engaged in the program by replying to messages after their quit date had passed.

The keywords used by the largest subset of participants were REASONS (11/20, 55%), CRAVE (10/20, 50%), and TIPS (7/20, 35%). Also of interest was the use of the keyword DATE—almost half (9/20, 45%) of the participants requested to reset their quit date mid-program by texting the keyword to the program. No participants used the keyword STOP to unsubscribe from the program.

**Table 3:** Program engagement and characteristics of responses to text messages.

Category and item^a^ (n=20)			n (%) or mean (SD)
**Overall engagement**			
		Read all texts received,^b^ n (%)	16 (100)
		Average total number of responses,^c^ mean (SD)	5.4 (6.6)
		Average response period in days, mean (SD)	24.2 (17.0)
		Participants who replied after their quit date, n (%)	12 (60)
**Survey and keyword use**			
	**Prequit surveys, n (%)**		
		Prequit smoking tracker	18 (90)
		Are you ready to quit (on quit date)?	14 (70)
		Are you ready to quit (before quit date)?	12 (60)
		Are you smoke free?	9 (45)
	**Postquit surveys, n (%)**		
		Postquit status tracker	10 (50)
		Pledge to stay smoke free	3 (15)
	**Anytime keywords, n (%)**		
		REASONS	11 (55)
		CRAVE	10 (50)
		DATE	9 (45)
		TIPS	7 (35)
		Requested a keyword GUIDE	7 (35)
		STATS	6 (30)
		SLIP	5 (25)
		SMOKED	5 (25)
		WHYQUIT	3 (15)
		GAME	1 (5)
		STOP	0 (0)

^a^Measures were collected from Voxiva programmatic records.

^b^Measures were collected at the 2-week follow-up (n=16).

^c^A response includes a reply to a text survey or an unsolicited request for support with quitting via text (eg, CRAVE).

### Self-Efficacy and Smoking-Related Outcomes

Participants were asked to rate their confidence in their ability to quit smoking while pregnant. At baseline, the average confidence rating was 3.6 (SD 1.2), demonstrating above-average levels of confidence in their ability to quit. At the 2-week follow-up, confidence levels rose to 3.8 (SD 1.3), and at the 4-week follow-up, confidence had risen to 4.8 (SD 0.5).

At baseline, participants smoked an average of 7.6 (SD 4.9) cigarettes per day. At the 2-week follow-up, the average number of cigarettes smoked had decreased to 4.7 (SD 5.2). At the 4-week follow-up, this number had decreased to 2.4 (SD 1.8) cigarettes per day. At the 2-week-follow-up, 5 participants out of 13 (38%) had reported abstaining for the past week, and 7 participants out of 13 (54%) reported abstaining for the past week at the 4-week follow-up.

## Discussion

### Principal Findings

Overall, we found support for the feasibility and acceptability of the Quit4baby program, with most participants agreeing that they liked the program overall. Participants overwhelmingly agreed that the texts were helpful in getting them to try to quit, that the texts gave them ideas on how to quit, and that they would recommend the program to a friend. Readership of the texts was high and sustained over time. The positive responses—plus the lack of negative responses—imply that the program was generally well liked and congruent with the target population’s needs.

It was encouraging that many participants used the interactive features of the text messaging system, a prominent feature of the program. Almost all participants initiated/replied to a text message and, on average, participants sent in more than 5 responses over the program period. While health-promotion programs that stimulate interaction and engagement have been found to be more likely to result in behavior change [29], it remains to be seen to what degree engagement in this program will be associated with smoking cessation.

As in previous studies [18], a significant proportion of participants stopped responding to the system by text once their quit date arrived. This finding likely reflects the fact that many participants did not follow through with their chosen quit date or quickly relapsed and then disengaged from the program as messages arrived, giving the erroneous impression that they had indeed quit smoking. It is possible that participants were hesitant to report relapse due to a variety of negative emotions, such as embarrassment or guilt. However, it is somewhat encouraging that the level of disengagement was lower than in previous studies and that numerous participants reset their quit dates. Still, the program could be redesigned to better engage such participants.

Pregnant smokers are hard to reach and have been reluctant to participate in offered programs. Since the Quit4baby program is being designed as an add-on service to Text4baby, it is hoped that the high reach of Text4baby and the offer of confidential, automated self-enrollment will provide a way to extend services to this hard-to-reach audience. This study helps demonstrate the plausibility of recruiting from Text4baby, one of the largest text messaging programs in the US [30], and concurrently offering an add-on, quit-smoking text messaging service. Most participants in our study—who continued to receive Text4baby messages while enrolled in Quit4baby—expressed that the programs fit well together. This is encouraging and validates the design plans. Though our enrollment rates were low, it is hoped that in future programs that directly screen for smoking and use automated systems, enrollment rates would increase.

Additional strengths of this study include that it involves the testing of a novel text messaging system for an at-risk and underserved population that makes use of interactive and personalized text messages. While the study sample was small, the pilot benefited from a follow-up rate of 80% (16/20) at the 2-week follow-up and 65% (13/20) at the 4-week follow-up.

### Limitations

Weaknesses of this study include the lack of a control group and that participation may have been limited by some Text4baby subscribers’ unwillingness to disclose their smoking activity. Due to low response rate (less than 3% response rate for all potentially eligible Text4baby subscribers), small sample size, and unique demographics of the sample (ie, 50% were single and 10% were married), the results are not generalizable to all pregnant women smokers. Another potential limitation includes the possible inflation of the DATE keyword engagement as GW research staff, in an estimated 2 cases, counseled participants on how to enter this command during the follow-up phone surveys. Once this practice was noted, it was discontinued.

### Conclusions

Findings show that a text messaging system that makes use of interactive and personalized text messages is acceptable to pregnant smokers enrolled in Text4baby. Insights gained from this study have informed the redesign of Quit4baby for a larger study and for possible dissemination. Given the emerging evidence for the efficacy of text messaging for smoking cessation [14], it is recommended that future text messaging studies strive to understand the utility of such programs for priority subgroups like pregnant smokers.

## Abbreviations

ASE: Attitude-Social Influence-Efficacy Model
FTND: Fagerstrom Test for Nicotine Dependence
GW: George Washington University
IRB: Institute Review Board
NRT: nicotine replacement therapy
SIDS: sudden infant death syndrome
SMS: short message service
